# Evaluation of genetic diversity among olive trees (*Olea europaea* L.) from Jordan

**DOI:** 10.3389/fpls.2024.1437055

**Published:** 2024-08-06

**Authors:** Mazen A. Al-Kilani, Francesca Taranto, Nunzio D’Agostino, Cinzia Montemurro, Angjelina Belaj, Salam Ayoub, Randa Albdaiwi, Shireen Hasan, Ayed M. Al-Abdallat

**Affiliations:** ^1^ National Center for Agriculture Research (NARC), Amman, Jordan; ^2^ Institute of Biosciences and Bioresources, National Research Council (CNR-IBBR), Bari, Italy; ^3^ Department of Agricultural Sciences, University of Naples Federico II, Portici, Italy; ^4^ Department of Soil: Plant and Food Sciences (DiSSPA), University of Bari Aldo Moro, Bari, Italy; ^5^ Centro “Alameda del Obispo”, Instituto Andaluz de Investigación y Formación Agraria, Pesquera, Alimentaria y de la Producción Ecológica (IFAPA), Córdoba, Spain; ^6^ Department of Allied Medical Sciences, Zarqa University College, Al-Balqa Applied University, Al-Salt, Jordan; ^7^ Hamdi Mango Center for Scientific Research, The University of Jordan, Amman, Jordan; ^8^ Department of Horticulture and Crop Science, School of Agriculture, The University of Jordan, Amman, Jordan

**Keywords:** genetic variation, center of domestication, molecular markers, olive, phylogenetic analysis, population structure, single nucleotide polymorphisms

## Abstract

This study aimed to identify and evaluate the genetic diversity of olive trees in Jordan, a country located in the eastern Mediterranean, where olive domestication originated. For this purpose, a total of 386 olive trees were analyzed, including 338 collected from two surveys (JOCC-1 and JOCC-2) across seven regions, and 48 selected accessions from the Olive Germplasm Bank of Jordan (JGBOC). These trees underwent comprehensive phenotypic and molecular characterization using different tools. Significant differences in morphological traits were detected among tested regions using the *Chi*-square test. Principal components analysis revealed that fruit color change and growth habit as the most discriminating traits, segregating the trees into two groups, with the first group including the Kanabisi cultivar and the second group including the Kfari Baladi cultivar. Utilizing Kompetitive Allele Specific PCR assay, two sets of informative SNPs were used for the genetic diversity analysis. Cladograms were constructed using the maximum likelihood method, revealing a consistent pattern where two clades containing identical genotypes were observed to cluster with the Kfari Baladi or Kanabisi. In addition, the SNP data was used to perform a comparative analysis with the Worldwide Olive Germplasm Bank of Córdoba, which revealed 73 unreported olive genotypes from Jordan. Genetic structure analyses using Discriminant Analysis of Principal Components (DAPC) identified four clusters with distinctive patterns of relatedness among 149 unique accessions, including 52 olive accessions from various Mediterranean countries (IOCC-3). ADMIXTURE analysis revealed four genetic clusters, consistent with the clustering observed in DAPC and cladogram analysis, indicating a high level of genetic admixture among Jordanian olive germplasm. In conclusion, the results show that olive trees in Jordan are highly diverse, providing valuable information for future conservation and management plans.

## 1 Introduction

The olive tree (*Olea europaea* L.) is a fruit tree native to the Mediterranean region that belongs to the Oleaceae family (Besnard, 2016). It has become an economically important crop, with approximately 10.95 million hectares cultivated globally with an estimated production of 18.40 million tons of olives (FAOSTAT, 2022). The Mediterranean region hosts 95% of the world’s cultivated olive growing area, with Spain (21.41%), Turkey (16.19%) and Italy (11.74%), as the main producers (FAOSTAT, 2022). It also has cultural and historical significance and is considered a sacred tree in various civilizations and one of the oldest domesticated plants in the world (Green, 2002).

The cultivated olive tree (*O. europaea* subsp. *europaea*) is believed to have originated from its wild ancestor, oleaster (*O. europaea* subsp. *sylvestris*), in the eastern parts of the Mediterranean basin (Barazani et al., 2023). Fossil pollen datasets obtained across the Mediterranean basin showed that the southern Levant served as the primary site for olive cultivation as early as ~6500 years BP, followed by a subsequent domestication process in Crete/Greece (Langgut et al., 2019). This indicates that the eastern Mediterranean basin is the primary center of olive domestication and is considered a natural habitat for wild and cultivated olive species (Julca et al., 2020). However, the history of olive domestication remains complex and somewhat mysterious, with archaeological and genetic studies suggesting several possible origins (Besnard et al., 2013; Lavee and Zohary, 2011). It remains unclear whether olive cultivars originated from a single initial domestication event in the Levant, followed by secondary diversification (Gros-Balthazard et al., 2019), or if the cultivated lineages resulted from multiple, independent primary domestication events (Jiménez-Ruiz et al., 2020).

In Jordan, a country situated in the eastern parts of the Mediterranean basin, olives are highly believed to have been domesticated as early as 6200 BCE, with evidence of oil pressing dating back to at least 5200 BCE in Pella, located in the northern Jordan valley (Dighton et al., 2017). During this period, the size and shape of olive endocarps changed, indicating a selection process for larger, more uniform olives fruits. An archeological study conducted by Jordanian and French scientists suggests that Hadeib Al-Reeh, a village in Wadi Rum, may be the oldest site in the world where olive tree cultivation dates back more than 7,500 years ago (Al-Shdiefat et al., 2012). Analysis of ashes from fireplaces in the village ruins revealed that olive cultivation dates back to the Chalcolithic period (around 5400 BC). This evidence strongly suggests that Jordan lies within the primary center of olive domestication in the Mediterranean region (Barazani et al., 2023).

To identify olive cultivars, scientists rely on morphological, physiological, and biochemical traits, as well as more recently on DNA molecular markers technologies (Boucheffa et al., 2019). Molecular tools have been used for olive cultivar identification, fingerprinting, and determining genetic relatedness patterns among them (Sebastiani and Busconi, 2017; Kaya et al., 2019; Marchese et al, 2023). Recently, new techniques based on next-generation sequencing technologies such as genotyping-by sequencing (GBS), double digest restriction-site associated DNA (ddRADseq), and diversity arrays technology (DArTseq) have enabled the generation of thousands of single nucleotide polymorphisms (SNPs). These SNPs have been used for cultivar characterization, analysis of genetic diversity, and have significantly enhanced our understanding of the genetic makeup of olive trees (D’Agostino et al., 2018; Kaya et al., 2019; Zhu et al., 2019; Islam et al., 2021; Slobodova et al., 2023). SNP markers have also proven to be an excellent tool for verifying the authenticity of table and oil olives (Ben Ayed and Rebai, 2019; Piarulli et al., 2019). Such new technologies have empowered genetic studies on the olive tree, enabling gene discovery, mapping of quantitative trait loci (QTL) and a deeper understanding of its domestication process (Koubouris et al., 2019; Mariotti et al., 2020).

In Jordan, the predominant olive cultivars include Nabali Baladi, Nabali Muhassan, and Rasie (Brake et al., 2014). However, numerous clones of these cultivars are dispersed throughout the country, resulting in the assignment of various common names. These names often differ based on the specific region where the olives are grown. The Nabali Baladi cultivar is regarded as one of the oldest olive cultivars in the Levant, believed to have originated on the banks of the Jordan River. It is favored by many farmers for its robust resilience and adaptation to dry environments, as well as its resistance against various pathogens (Brake et al., 2014). Furthermore, in Jordan, farmers continue to utilize centuries-old olive trees known as Romi, a term referring to the era of the Romans. These trees grow naturally in various regions across Jordan and are preferred over introduced cultivars from foreign origins (Brake et al., 2014). The centenary age of the Romi trees, their adaptation to harsh environment, resilience to various stresses, and high quality of their extracted oil indicate a rich genetic diversity. This highlights their significance and emphasizes the urgency of conserving and integrating them into the local olive production system.

One of the primary challenges facing the cultivation of olive trees in Jordan is the limited understanding of their origins, degree of genetic purity and diversity, particularly whether they are considered clones or not. Therefore, scientists are actively investigating the genetic relationships between wild and cultivated olive trees that naturally thrive in various parts of the country. The second major problem is the loss of such valuable genetic material from its natural habitat, exacerbated by climate change, urbanization, and insufficient awareness of their historical importance (Belaj et al., 2016). Furthermore, such material, growing naturally in remote and marginal areas, is often neglected, lacking proper care and good agricultural practices that are crucial for optimal growth and production. Therefore, it is important to shed the light on the genetic diversity of olive germplasm in Jordan, a country where olive cultivation is believed to have originated. This will aid in better understanding their importance and underscore the necessity of developing action plans to preserve them using both *in situ* and *ex situ* approaches.

This study assesses the extent of genetic diversity among Jordanian olive trees using molecular markers and morphological traits, with the aim of guiding olive conservation and management strategies, especially in the context of climate change and genetic erosion. Understanding the genetic variability of Jordan olive trees in arid conditions may facilitate the identification of genes/alleles to be introduced in olive breeding programs across the Mediterranean region. To this end, two targeted prospecting surveys were conducted across Jordan to identify olive trees growing across different habitats. The identified trees were characterized by different morphological traits, which were used to categorize them into distinct sets. The genetic identity and level of variation among olive genotypes were assessed with a selected set of SNP markers through the KASP (kompetitive allele-specific PCR) genotyping assay. The obtained results provide valuable insights into the distribution and characteristics of olive trees across different regions of Jordan, enhancing our understanding of the genetic diversity within the country’s olive tree heritage and their ecological significance. The data highlights the importance of Jordanian olive genetic resources and emphasizes the pressing need for future conservation plans, innovative propagation strategies, and integration into the national olive production system.

## 2 Materials and methods

### 2.1 Plant material, survey procedure and description of the collection sites

In this study, two field surveys were conducted in 2020 and 2022 across various regions of Jordan to identify and characterize olive trees (**Table 1**; **Supplementary Figure S1**). Both surveys spanned from April to December and targeted olive trees older than 100 years, estimated by measuring the trunk diameter at 130 cm from the ground following the procedure outlined by Arnan et al. (2012). Comprehensive information, including site description (regions representing governorates and areas representing municipalities within each governorate) with respective geographic coordinates (latitude, longitude, elevation), physiography, habitat, micro-climate conditions, human management practices, density, and distribution, was collected for each Jordanian locality (**Supplementary Table S1**). Additionally, meteorological data such as average annual rainfall, temperature, and humidity were obtained from the nearest meteorological station for each site, sourced from the Jordanian Meteorological Department (data not shown).

**Table 1:** Collection, locations, regions, area names, climatic zones, and the number of sampled trees in the study.

Collection	Location	Region	Area	Number of Sampled Trees	Climatic Zone
**JOCC-1**	North of Jordan	Ajloun	Ain Janna	6	Mediterranean sub-humid
		Ajloun	Al Hashimiyya	3	Mediterranean semi-arid
		Ajloun	Anjara	3	Mediterranean sub-humid
		Ajloun	Khirbat al Wahadinah	13	Mediterranean semi-arid
		Ajloun	Kufranjah	7	Mediterranean semi-arid
		Irbid	Al Taybeh	12	Mediterranean semi-arid
		Irbid	Al-Korah	12	Mediterranean semi-arid
		Irbid	Al-Mazar-N	12	Mediterranean sub-humid
		Irbid	Bani Kananeh	14	Mediterranean semi-arid
		Irbid	Kufr-Asad	12	Mediterranean semi-arid
		Jarash	Al-Kittah	18	Mediterranean semi-arid
		Jarash	Borma	15	Mediterranean semi-arid
		Jarash	Kausheba	6	Mediterranean semi-arid
	Central of Jordan	Balaqa	ArRumaymin	2	Mediterranean sub-humid
		Balaqa	Buyoida	2	Mediterranean semi-arid
		Balaqa	El-Baque	4	Mediterranean sub-humid
		Balaqa	Maysarah	9	Mediterranean semi-arid
		Balaqa	Shafa-Badran	5	Mediterranean semi-arid
		Balaqa	Umm-Joza	2	Mediterranean sub-humid
	South of Jordan	Karak	Aiy	7	Mediterranean semi-arid
		Karak	Al Taybeh-S	7	Mediterranean arid cool
		Karak	Iraq	2	Mediterranean arid cool
		Ma’an	Megaraeh	4	Mediterranean arid cool
		Ma’an	Wadi Musa	8	Mediterranean arid cool
		Ma’an	Wadi Rum	2	Saharan Mediterranean
		Tafilah	Aema	7	Mediterranean semi-arid
		Tafilah	EL-Balad	3	Mediterranean semi-arid
		Tafilah	El-Meshref	3	Mediterranean semi-arid
**JOCC-2**	North of Jordan	Jarash	Borma	44	Mediterranean semi-arid
	Central of Jordan	Balaqa	ArRummain	16	Mediterranean semi-arid
		Balaqa	Buyoida	13	Mediterranean semi-arid
		Balaqa	Maysarah	49	Mediterranean semi-arid
		Balaqa	Shafa-Badran	4	Mediterranean semi-arid
		Balaqa	Umm Jozeh	12	Mediterranean sub-humid
**JGBOC**	Central of Jordan	Madaba	Gene Bank	48	Mediterranean semi-arid
**IOCC-3**	Italy	Taranto	Palagiano	52	Mediterranean humid

In the first prospecting survey (Jordan olive core collection-1; JOCC-1), a total of 200 olive trees were sampled from seven different regions: Irbid (IR) (n. = 62), Jarash (JA) (n. = 39), Ajloun (AJ) (n. = 32), Balaqa (BA) (n. = 24), Al-Karak (KR) (n. = 16), At-Tafilah (TF) (n. = 13), and Ma’an (MN) (n. = 14). In the second prospecting survey (Jordan olive core collection-2; JOCC-2), two regions from the first survey, namely BA and JA, were extensively studied where both regions were re-targeted based on preliminary observations of significant morphological variation and abundant genetic diversity observed during the molecular analysis of olive trees sampled in the initial prospecting survey. A total of 138 olive trees were sampled in the second prospecting survey, including 94 trees from BA and 44 from JA. Additionally, this study included 48 cultivated varieties from the Olive Germplasm Bank of Jordan (Jordan Gene Bank Olive Collection (JGBOC), located in Al-Mushagar (NARC, Jordan), bringing the total number of Jordanian olive trees under study to 386 (**Supplementary Table S1**). Finally, 52 trees representing olive accessions from various Mediterranean countries were selected from the open-field collection (Italy Olive Core Collection (IOCC-3)) conserved in Palagiano, Taranto, Italy (**Supplementary Table S1**) and were used for the genetic structure and relationships analysis.

In this study, each recorded Jordanian olive tree received a unique identification code to specify its origin and collection site (**Supplementary Table S1**). For this purpose, the 386 trees of Jordan were grouped based on their geographical region: IR (Irbid), AJ (Ajloun), JA (Jarash), BA (Balaqa), and KR (Karak), TF (Tafilah), MN (Ma’an), while the last group included accessions retrieved form JGBOC (**Supplementary Table S1**).

### 2.2 Morphological characterization

The morphological characterization involved the examination of 28 traits following the methodology outlined in Barranco et al. (2000). The analysis included both the olive trees sampled during the field surveys (334 trees, with data missing for four trees) and the 48 JGBOC accessions. A complete list of the studied morphological traits, along with descriptions, measurement methods, and recorded data are given in **Supplementary Table S2**. In addition to the traits outlined in Barranco et al. (2000), measurements of trunk diameter (Arnan et al., 2012) and fruit color change location (starting point of fruit coloration: apex; uniform; base) were included. Morphological data were subjected to statistical analysis using the *Chi*-square test based on the region of origin of the trees, as described above (**Supplementary Table S1**).

To explore patterns of variation in morphological traits among individuals, Principal Component Analysis (PCA) was performed using ‘FactoMineR’ version 2.4 (Lê et al., 2008; https://cran.r-project.org/web/packages/FactoMineR/index.html) and ‘factoextra’ version 1.0.7 (Kassambara, 2017; https://cran.r-project.org/web/packages/factoextra/index.html). To visualize clustering patterns within the dataset, a hierarchical cluster was built using the ‘pheatmap’ package v1.0.8 in R (Kolde and Kolde, 2015; https://cran.r-project.org/web/packages/pheatmap/index), using Euclidean distances and the Ward.D2 method.

Additionally, a heatmap was generated, that visually represents the morphological data of the 382 olive trees. Furthermore, Pearson’s correlation coefficients were calculated using the ‘corrplot’ package in R (Wei et al., 2017; https://cran.r-project.org/web/packages/corrplot/vignettes/corrplot-intro.html) to quantify the relationships and correlations between various morphological traits.

### 2.3 Molecular characterization

Total genomic DNA (gDNA) was extracted from fresh young leaves from the canopy of each olive tree using the Wizard^®^ Genomic Purification Kit (Promega, Madison, WI, USA) following the manufacturer’s instructions. The quantity and quality of the extracted gDNA were assessed using a 1% agarose gel stained with Red Safe (Intron, Bio-tek, Seoul, Korea) and a spectrophotometer (BIO-RAD, SmartSpecTM Plus, Hercules, CA, USA). Subsequently, a gDNA stock solution (30 ng/µL) was prepared for each sample using sterile distilled water and stored at -20°C until further analysis.

Two sets of SNP markers were used for KASP analysis (**Supplementary Table S3**). KASP technology was selected for its ability to specifically target individual SNP loci with high accuracy. It offers cost-effectiveness for large-scale studies, scalability across hundreds to thousands of SNP markers, and consistent reproducibility across different laboratories. Additionally, its compatibility with automation enhances workflow efficiency and minimizes potential human errors. The SNP markers in both sets were selected based on their highest degree of authenticity score. The first set of markers included 24 SNPs sourced from two previous studies: Biton et al. (2015) (N. = 12) and Belaj et al. (2018) (N. = 12). This set was successfully tested on 173 trees from JOCC-1, along with 42 trees from JGBOC. The second set included 48 SNPs from Belaj et al. (2022) and was tested on 203 trees, encompassing 132 trees from JOCC-2, 37 selected trees from JOCC-1, and 34 accessions from JGBOC.

The SNP profile from the second set was utilized for two primary objectives. Firstly, it was compared with the World Olive Genebank Collection (WOGBC) database to evaluate the reliability of the EST-SNP markers across various laboratories and techniques, and to determine how the genetic profile or SNP markers of olive trees from Jordan compare or match with those already recorded in the WOGBC database (Belaj et al., 2022). Secondly, the same set of markers was employed for conducting the Discriminant Analysis of Principal Components (DAPC) and population structure analysis. This analysis involved 52 trees from the IOCC-3 and 203 olive trees from Jordan, as described previously.

The SNP marker sequence data was used to design KASP assays, which were performed and analyzed by LGC-Genomics (Hoddesdon, UK). For the first and second sets of SNP markers, 22 and 45 assays were successfully designed and tested, respectively. The genotypic data obtained from these assays were analyzed using LGC’s KlusterCaller software (https://www.biosearchtech.com/products/pcr-reagents-kits-and-instruments/pcr-instruments-and-software/genotyping-and-lims-software/klustercaller-genotyping-software) and visualized using LGC’s SNP-viewer software (https://www.biosearchtech.com/products/pcr-reagents-kits-and-instruments/pcr-instruments-and-software/genotyping-and-lims-software/snpviewer). Each marker was scored based on its allele call in each individual, and the results were transformed into a binary matrix. In this matrix, the presence of homozygous alleles was scored as 00 or 11, while heterozygous alleles were represented as 01.

### 2.4 Genetic diversity and population structure analysis

The heterozygosity index (H), polymorphism information content (PIC), and discriminating power (D) were calculated using iMEC (https://irscope.shinyapps.io/iMEC/). SNP data were used to construct cladograms using the maximum likelihood method (with default parameters under the BINGAMMA substitution model), with 1,000 bootstrap replicates using RAxML (Randomized Axelerated Maximum Likelihood) (Edler et al., 2021). The constructed tree was refined and visualized using the Interactive Tree of Life online tool (Letunic and Bork, 2021). PLINK v.1.90 (Purcell et al., 2007) was used to compute the IBS (Identical-By-State) distance matrix between pairs of accessions using the second set of markers. Identical genotypes within different regions (JA: Borma and BA: Maysarah, ArRummain, Byouida and Umm Jozeh) were found by setting an IBS value ≥ 0.95 (to capture as much intra-region variability as possible).

After removing duplicates within regions, a more stringent IBS analysis was conducted with a threshold of ≥ 0.98 to search for duplicates in the JOCC-1, JOCC-2 and JGBOC collections and reduce putative redundancy. Only one tree per unique genotype was retained for downstream analyses. Population structure was investigated using two different approaches. Allele frequency and ancestry estimation were performed using ADMIXTURE v. 1.3.0 (Alexander and Lange, 2011), with 10-fold cross validation (CV) for subpopulations (K) between 1 and 15, and 1,000 bootstrap replicates. CV scores were used to estimate the optimal K value. A membership coefficient (q_i_) > 0.55 was used to assign individuals to each cluster. A DAPC analysis was performed using the ‘adegenet’ package in R (Jombart et al., 2010; Jombart and Ahmed, 2011). The optimal number of principal components (PCs) was determined using a value ≥ 1:200. The optimal number of subpopulations was assessed using the Bayesian information criterion (BIC). Pairwise genetic distance between subpopulations identified by DAPC was estimated using the Weir and Cockerham’s average *F*_ST_, implemented in SVS v.8.9.1.

## 3 Results

### 3.1 Occurrence of olive trees in Jordan

**Figure 1:**
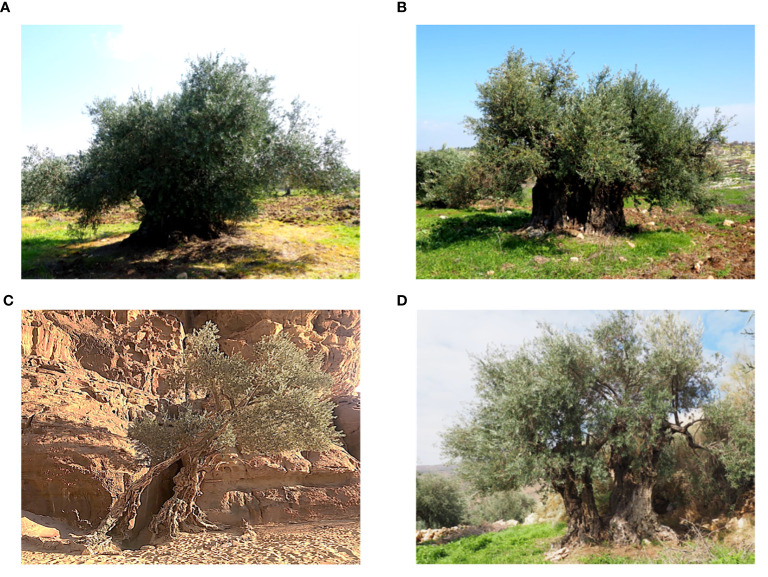
Olive trees from selected areas of Jordan **(A)** Irbid, **(B)** Ajloun, **(C)** Wadi Rum and **(D)** Karak.

In this study, two surveys were conducted to prospect and identify olive trees in their native range and on farmers’ fields across seven distinct regions in Jordan, encompassing a total of 28 selected areas (**Table 1**; **Supplementary Figure S1**; **Supplementary Table S1**). Thus, 388 olive trees were sampled. These trees were located at altitudes ranging from 166 m in Khirbat Wahadinah to 1220 m in Wadi Musa. The regions targeted in this study had an average annual rainfall between 50 mm and 586 mm, with the highest recorded in AJ and the lowest in Wadi Rum. The number of trees varied depending on the region examined, with the highest concentration of olive trees found in northern Jordan, where the density of olive trees is considerably high (**Supplementary Figure S1**; **Supplementary Table S1**). The collected trees were found in 28 areas (municipalities) of the seven targeted regions (governorates), with the numbers of trees per area ranging from two (Wadi Rum (MN)) to 59 (Borma (JA)). The olive trees were found growing in nature (hillsides, hilltops, rock face, forests, rangelands, forests and mountains) or in cultivated fields and were in various topographies, with the majority in the plains (**Figure 1**; **Supplementary Table S1**). The predominant habitat of the sampled trees was arable lands, with two trees identified in the Wadi Rum desert. Most olive trees were found in managed farmers’ fields, while 41 trees grew naturally in remote areas. In southern areas of the country olive trees thrived and remained productive thanks to supplementary irrigation practices by local farmers, despite the dry conditions. In contrast, northern areas relied predominantly on rainfed production systems. Despite drought stress from precipitation fluctuations, trees in these regions continued to grow and produce satisfactorily.

### 3.2 Morphological data

Data analysis indicated significant differences in several morphological traits, as confirmed by the *Chi*-square test using phenotypic data for 382 trees across eight identified regions (**Supplementary Table S4**). Out of the examined traits, 24 showed statistically significant differences (*p ≤ 0.05*). Traits related to the presence and size of lenticels and color at full maturity did not exhibit significant differences and were excluded from downstream analyses. Significant differences were observed in fruit weight among olive trees, with notable frequencies across different regions. Specifically, trees from the JA region showed the highest frequency (51.61%) of “low weight” fruits, while JGBOC accessions exhibited the highest frequency (87.50%) of “the very high weight” fruits (**Supplementary Table S4**). These results indicate distinctive variations in morphological characteristics among the olive trees, providing valuable insights into their diversity and potential genetic differentiation.

**Figure 2:**
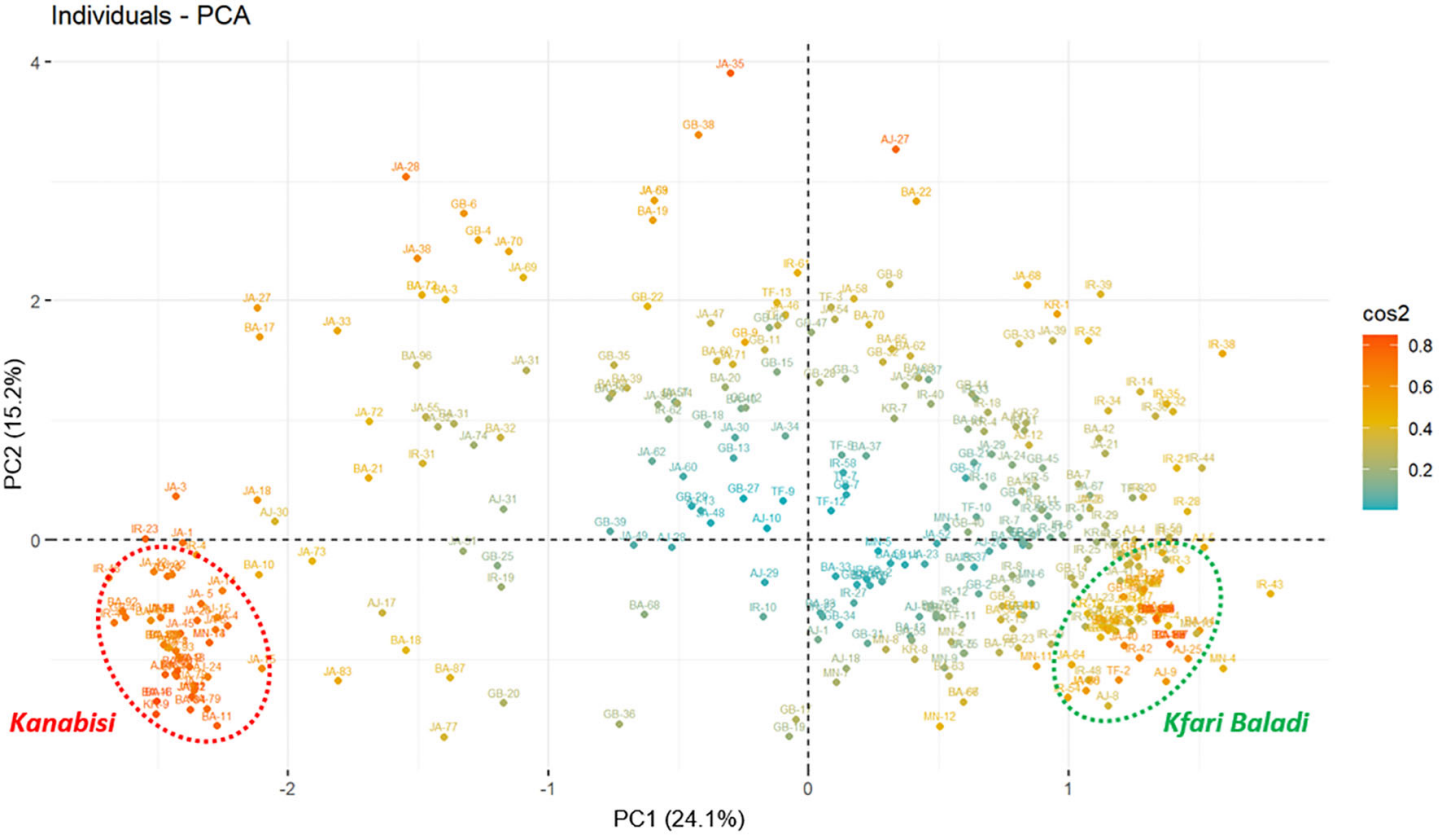
Principal component analysis (PCA) plot based on morphological traits measured for 382 olive trees showing the distribution of the of 382 olive trees (cos2: quality of the representation of individuals based on the principal components).

To analyze the most discriminating traits among the defined groups, principal components analysis (PCA) was performed. The Eigenvalues of the top 10 principal components (PCs) explained 78.01% of the total variation, with PC1 explaining 24.10% and PC2 contributing 15.20% (**Figure 2**; **Supplementary Figure S2**). Among the variables tested, the largest contribution in the PCA was observed for traits related to the location of the color change and growth habit. These traits clustered closely together in in quadrant IV of the PCA plot, distinctly separated from the other traits (**Supplementary Figure S2**). Moreover, “stone termination” was loaded separately in quadrant II of the PCA plot, indicating its potential role in discriminating among the identified olive trees. Finally, several traits were found to be highly correlated in the PCA, such as fruit and stone diameter, fruit apex with stone base, tree vigor, fruit weight, fruit symmetry, and stone symmetry (**Supplementary Figure S2**). Overall, the PCA identified key morphological traits that significantly contribute to differentiation and genetic variation among the olive tree populations under study.

The PCA results depicted a clear separation of the sampled trees into two distinctive groups (**Figure 2**). The first group was positioned in quadrant IV and included a JGBOC accession known as Kanabisi, along with several olive trees collected from different sites across the country that were also recognized by local farmers as Kanabisi. The second group was positioned in quadrant III of the PCA plot and included a JGBOC accession known as Kfari Baladi, along with several other accessions from JGBOC (such as Nabali Baladi, Kfari Baladi, Arabi Tafilah, Ketat, etc.) and various olive trees collected from different sites across the country, which were recognized by local farmers as Romi or Baladi. Out of the two defined groups, most of the remaining trees were either positioned in quadrant III near to the Kfari Baladi group or randomly scattered, indicating a higher degree of phenotypic diversity (**Figure 2**). This set included several trees identified in survey 2 (JA and BA regions) as well as several accessions retrieved from JGBOC.

As mentioned above, several morphological traits can act as discriminators between Kfari Baladi and Kanabisi olive genotypes. These traits include growth habit, stone weight, fruit and stone diameter, site of initial fruit color change, longitudinal curvature of the leaf blade, stone termination, and stone base (**Supplementary Figure S2**). In terms of growth habit, trees of Kfari Baladi genotype exhibited a higher prevalence of the drooping growth habit, whereas the erect growth habit was more frequent in Kanabisi and trees that showed the same genotype with it. Kfari Baladi trees tended to show a drooping phenotype, characterized by slender shoots and branches that curve downward from the outset (**Supplementary Figure S3**). Conversely, the growth habit of Kanabisi trees was characterized by strong apical dominance, with branches tending to grow vertically, resulting in a crown that takes on a distinct conical shape, transitioning to cylindrical upon reaching maturity. Regarding the location of the onset of color change, the Kfari Baladi fruits showed the onset of color change from the base of the fruit, while the Kanabisi genotype showed the onset of color change from the apex of the fruit. For “the longitudinal curvature of the leaf blade”, Kfari Baladi trees predominantly exhibited an epinastic curvature type, whereas the flat curvature type was more prevalent in the Kanabisi trees. As for stone weight, the highest frequency of a “medium weight” score was noted in Kfari Baladi trees, whereas the frequency of “high weight” was more pronounced in the Kanabisi trees. These distinct morphological traits offer a basis for discriminating between the olive trees of Kfari Baladi and Kanabisi (**Supplementary Figure S3**).

**Figure 3:**
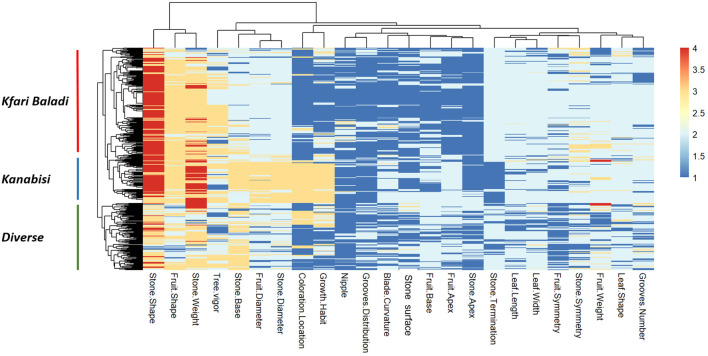
Heatmap and two-dimensional hierarchical clustering using data from 24 phenotypic traits measured in the 382 olive trees under study.

Significant positive and negative pairwise correlations (*p ≤ 0.05*) were observed between different traits (**Supplementary Figure S4**). Interestingly, a strong positive correlation was observed between growth habit and fruit diameter, stone diameter, and the location of coloration change, while stone termination showed a strong negative correlation with these traits. This is in general agreement with the PCA results (**Supplementary Figure S2**). The heatmap in **Figure 3** allows three distinct clusters to be distinguished based on genetic background that overlaid with the PCA clustering of Kanabisi, Kfari Baladi, and diverse (i.e., the set of individuals not included in any of the previous clusters). Overall, the analysis of morphological traits provided a complete overview of the relationships and existing variation among the olive trees under study. However, these results alone may not resolve the issue of redundancy among Jordan’s olive trees.

### 3.3 Molecular data analysis

Two sets of informative SNPs, originally derived from previous studies (Biton et al., 2015; Belaj et al., 2018, 2022), were converted into KASP assays to assess genetic diversity among the olive trees under investigation (**Supplementary Table S3**). The first set of markers was used to genotype 215 olive trees (comprising 173 trees from JOCC-1 and 42 trees from JGBOC). Unfortunately, 39 individuals were excluded from downstream analyses due to failures of the KASP assay. The second set of markers was used to genotype 203 olive trees (consisting of 132 trees from JOCC-2, 34 accessions from JGBOC, and 37 selected trees from JOCC-1). Only three olive trees failed to produce informative genotypic data and were consequently excluded from downstream analysis. The analysis of the KASP data from the first set revealed that the SNP marker *OLIVESNP191* exhibited the highest heterozygosity index (0.59) and polymorphic information content (PIC) (0.50) (**Supplementary Table S5**). Conversely, the lowest heterozygosity index (0.10) and polymorphic information content (PIC) (0.09) were observed for *OLIVESNP600*. For the second set, SNP marker *EContig5183* exhibited the highest heterozygosity index (0.55) and polymorphic information content (PIC) (0.45), while the lowest heterozygosity index (0.12) and polymorphic information content (PIC) (0.12) were observed for *EContig2937_4* (**Supplementary Table S5**).

**Figure 4:**
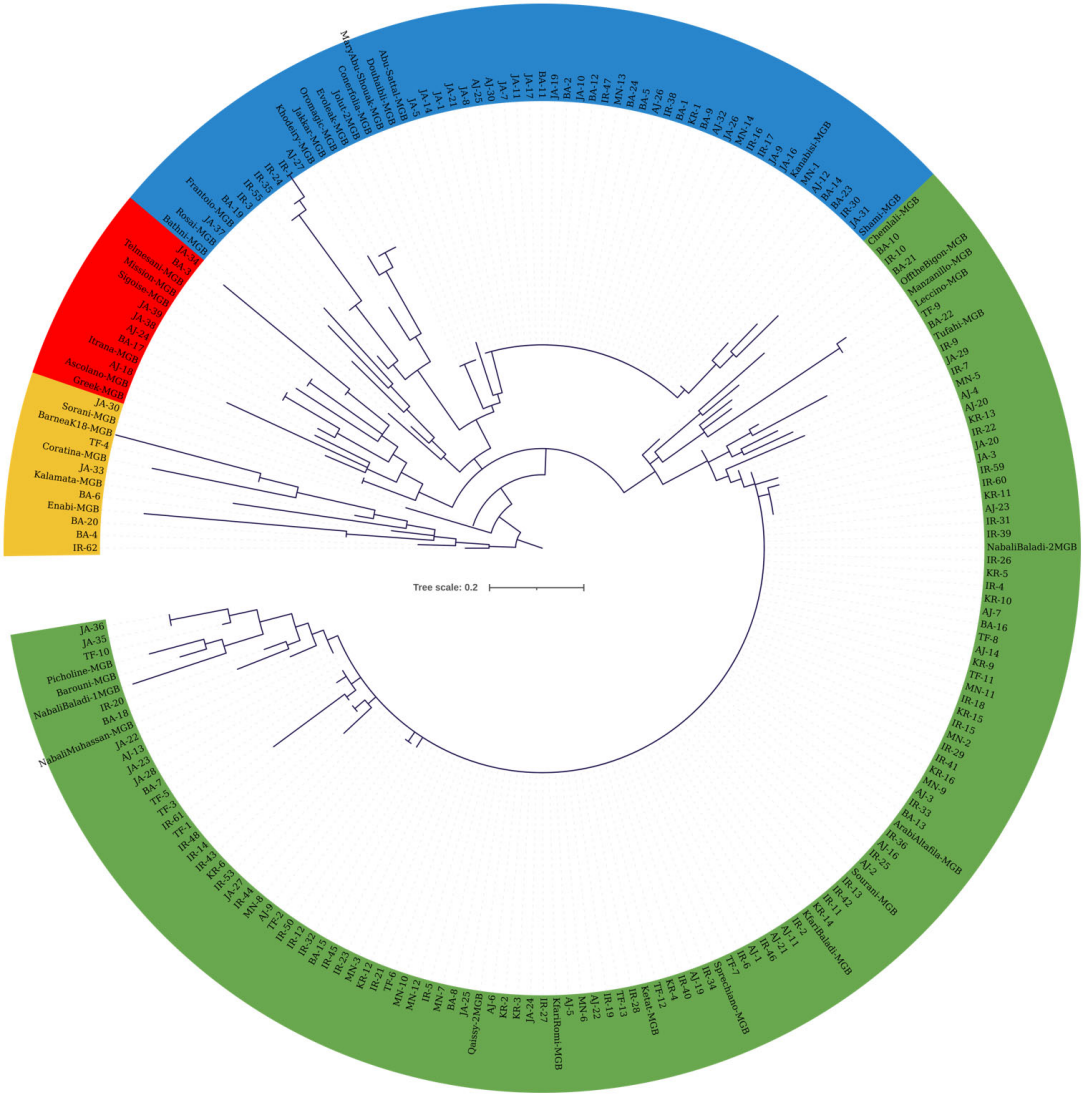
Cladogram constructed using the maximum likelihood method (bootstrapping value of 1,000) based on the genotyping data from 22 SNP loci. The analysis included 173 olive trees from JOCC-1 and 42 accessions from JGBOC.

Molecular marker data from both sets of SNPs were used to construct cladograms. The cladogram in **Figure 4** was generated using KASP data from 173 olive trees from JOCC-1 and 42 trees from JGBOC, which organized olive trees into four distinct clades. Clade 1 (yellow colored) comprised 12 genotypes, including five accessions from JGBOC (Barnea (K18), Coratina, Kalamata, Enaby and Sorani), and seven Jordanian trees (BA-4, BA-6, BA-20, IR-62, JA-30, JA-33, and TF-4). The second clade (green colored) accounted for 62.09% of the total olive genotypes (131 out of 211) and consisted mainly of Kfari Baladi trees. Thus, within this cluster, 89 trees were found to be very similar (as inferred by zero length branches) suggesting that Kfari Baladi is a master cultivar widely cultivated in various regions of Jordan. In addition, several accessions from JGBOC (Sourani, Kfari Baladi, Arabi Tafileh, Ketat, and Nabali Baladi-2) also shared the same SNP genotypes with Kfari Baladi. Two smaller groups were also identified within this cluster, the first including five olive trees (BA-18, IR-20, TF-10, and two similar trees, JA-35 and JA-36) grouped with four accessions from JGBOC (Nabali Muhassan, Nabali Baladi-1, Barouni, and Picholine) (**Figure 4**). The second group included five accessions from JGBOC (Chemlali Sfax, Off The Bigon, Manzanilla de Sevilla, Leccino and Tufahi) and eight olive trees from Jordan. The third clade (blue colored) included 34 trees that shared the same genotype with Kanabisi indicating thus the presence of another master cultivar grown predominantly in various regions of Jordan. Interestingly, the genotypes within clade-3 overlap with the ones identified as “Kanabisi” in the morphological analysis. Additionally, olive trees from the IR region (IR-1, IR-3, IR-24, IR-35, IR-55) grouped together, indicating close kinship between them (**Figure 4**). Within this clade, several accessions from JGBOC were found to be identical, indicating redundancy even in the gene bank material. The fourth clade (red colored) included six JGBOC accessions and seven trees, of which two (JA38 and JA39) were found to be identical (**Figure 4**).

**Figure 5:**
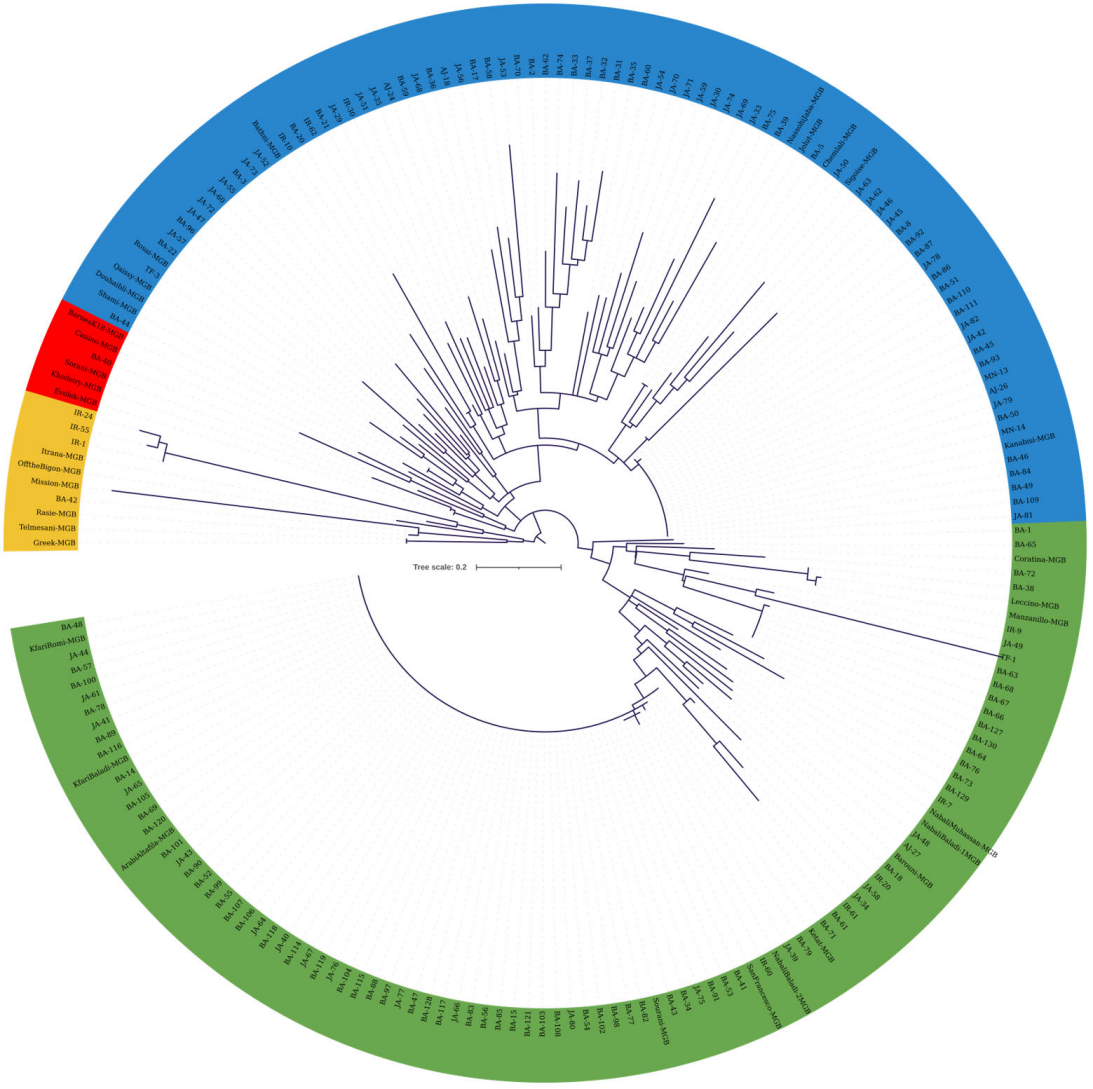
Cladogram constructed using the maximum likelihood method (bootstrapping value of 1,000) based on the genotyping data of 45 SNP loci. The analysis included 132 olive trees from JOCC-2, 34 olive trees from JOCC-1 and 34 accessions from JGBOC.

The cladogram in **Figure 5** was generated using KASP data from 45 SNP loci, allowing the distinction of four clades. Despite the increased number of markers, a significant portion of the collected material displayed 100% similarity, once again indicating the presence of redundant genotypes within the collection (**Figure 5**). The first clade (yellow colored) comprised 10 genotypes, including six JGBOC accessions, three trees from JOCC-1 (IR-1, IR-24, and IR-55), and a single tree from JOCC-2, which is identical to Rasie, a Jordanian cultivar. Notably, the three trees from JOCC-1 also grouped together in the cladogram of **Figure 4**. The second clade (red colored) comprised six genotypes, five of which were from JGBOC (Barnea (K18), Canino, Sorani, Khodeiry, and Evolek). This clade also included BA-40 collected in JOCC-2. The third clade (blue colored), the second largest clade (comprising 87 genotypes, i.e., 42.86% of the total), showed the highest diversity (**Figure 5**). This clade incorporated 10 accessions from JGBOC, all classified as Eastern Mediterranean cultivars with the exception of Sigoise. Among these, was the cultivar ‘Kanabisi’, which showed complete identity with 23 genotypes within the clade. The fourth clade (green colored) consisted mainly of Kfari Baladi trees, indicating the dominance of this genotype in different regions of Jordan (**Figure 5**). Several genotypes closely related to Kfari Baladi were observed within the same clade, including 11 trees and three JGBOC accessions (Barouni, Nabali Baladi-1, and Nabali Muhassan). Furthermore, the clade included a smaller subgroup of 16 genotypes, consisting of three JGBOC accessions (Manzanilla de Sevilla and Leccino, oddly identified as identical being thus a possible JBOC management error, and Coratina) and 13 genotypes, with 10 trees collected from the BA region, of which nine were from JOCC-2 (**Figure 5**). Interestingly, six trees (BA-63, BA-66, BA-67, BA-68, BA-127 and BA-130) were found to be identical, indicating the presence of a new and previously unknown cultivar uniquely adapted to the BA region.

### 3.4 Genetic structure and relationships of olive trees from Jordan and other countries

The KASP data obtained from the second survey (45 SNP markers) were used to confirm the identity of the Jordanian olive germplasm. They revealed a high reliability when compared with the EST-SNP profiles from the WOGBC database (Belaj et al., 2022). In addition, as expected and considering that the Jordanian plant material was provided from the JGBOC to the international olive collection of Córdoba, the data comparison between the two collections confirmed the redundant germplasm described above within the Jordanian collection. Besides, including a large WOGBC database of 668 different genotypes the comparison made possible to confirm previously known synonyms of Jordanian olive germplasm with cultivars from neighboring countries. Thus, as expected, the 62 olive trees that were identified as Kfari Baladi as well six JGBOC accessions (Kfari Romi, Arabi Tafilah, Ketat, Kfari Romi, Nabali Baladi-2 and Sourani) shared the same genotype with the Lebanon cultivar ‘Baladi’ and its synonyms (**Supplementary Table S6**). On the other hand, it was confirmed that 23 prospected olive trees shared the same genotype with Kanabisi, as shown previously (Belaj et al., 2022), that belongs to the synonymy group of the Syrian cultivar ‘Safrawi’. Interestingly, two olive trees collected from BA were genetically identical to two accessions from JGBOC. Specifically, BA-38 was identical to Leccino (ITA), which might be a collection mistake or sampling error, while BA-42 was identical to Rasie (synonymous with Nabali Muhassan (JOR), Gordal De Granada (ESP) etc.). The six unique trees that were found to be identical did not match with any cultivar from the WOGBC database, indicating the presence of unknown cultivated germplasm among the prospected trees in Jordan.

The analysis also revealed 14 possible errors of donor collections and/or mislabeling or propagation errors in the JGBOC, especially in the case of introduction of foreign plant material. For instance, the JBBOC cultivars ‘Manzanilla de Sevilla’ and ‘Coratina’, two important cultivars from Spain and Italy, respectively, showed different SNP profile with the same cultivars from WOGBC that were previously authenticated (Trujillo et al., 2014). Similarly, Sigoise, a Western Mediterranean cultivar from Algeria (synonymous of Picholine Marocaine; Belaj et al., 2022), was found to share the same SNP genotype with a totally different (by both morphological and molecular description at WOGBC) Greek cultivar named ‘Gaydoyrelia’ (**Supplementary Table S6**). This finding could explain its initial grouping with eastern Mediterranean varieties (**Figure 5**). Interestingly, the comparative analysis confirmed the presence of 73 new and unique genotypes among the prospected olive trees (**Supplementary Table S6**).

**Figure 6:**
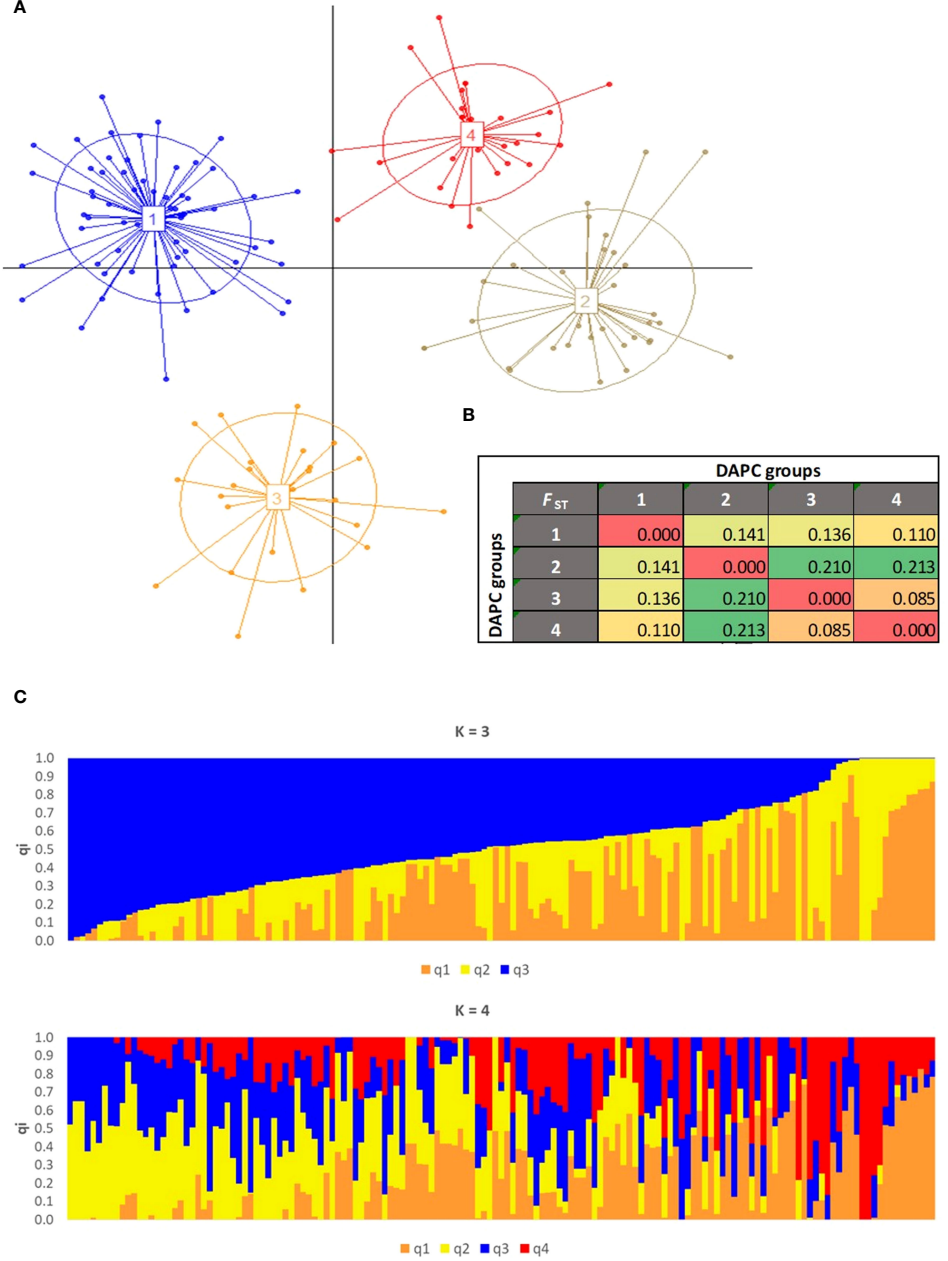
Population genetic structure assessed on the 149 olive accessions by **(A)** DAPC scatter plot. The axes represent the first two Linear Discriminants (LD). Each circle represents a cluster and each dot represents an individual. Numbers and colors refer to four different groups identified by BIC values. **(B)** Pairwise *F*_ST_ distance values between the four clusters identified by DAPC. **(C)** Population structure with 3 and 4 ancestries.

To eliminate the presence of duplicate and redundant olive genotypes in JOCC-2 and IOCC-3 (comprising 252 samples), an initial IBS analysis was performed within each sampling area of Jordanian trees. Using a threshold of IBS ≥ 0.95, 26, 34, 2, 1, and 2 individuals were retained for the Borma (43 samples), Maysarah (57 samples), Umm Jozeh (12 samples), Byouida (10 samples), and ArRummain (16 samples) groups, respectively. After removal of these duplicated or redundant genotypes, a subsequent IBS analysis (IBS ≥ 0.98) was carried out on the remaining 183 olive trees (**Supplementary Table S7**). A total of 34 duplicate individuals were identified and subsequently excluded from the dataset, resulting in a final collection of 149 unique olive genotypes that included 28 trees from JOCC-1, 48 trees from JOCC-2, 21 cultivars from JOGBC and 52 cultivars from IOCC-3. These 149 different genotypes were then used for the subsequent genetic structure analysis. To gain insights into the genetic structure, two separate analyses were performed (**Figure 6**). DAPC analysis identified four clusters of genetically related individuals (**Supplementary Figure S5**). Cluster 1 (C1) encompassed 70 individuals, predominantly from the Borma (n. = 21) and Maysarah (n. = 22) sites including few Mediterranean cultivars (**Figure 6A**; **Supplementary Table S8**). Cluster 2 (C2) consisted of genotypes (n. = 16) with different Jordanian origins and the two cultivars, Kfari Baladi and Kanabisi. Most olive cultivars from Italy and other Mediterranean countries were grouped into clusters 3 (C3) and 4 (C4), with a few Jordanian trees included in cluster 4 (IR-1, IR-24, IR-55, and BA-3). ADMIXTURE analysis identified three and four genetic pools (with cross-validation error values of 0.6627 and 0.6621, respectively) (**Figure 6B**, **Supplementary Figure S6**). At K = 3, a group of 26 Mediterranean cultivars was separated into the q1K3 gene pool, corresponding to cluster C3 in the DAPC analysis. While 16 genotypes belonging to the q2K3 gene pool largely overlapped with the DAPC C2 cluster. The q3K3 gene pool included genotypes with different origins, similar to the C1 cluster in DAPC, resulting in 44 trees that showed high admixture (**Supplementary Table S8**). At K = 4, it was observed that the gene pools q1K4 and q4K4 corresponded to Mediterranean cultivars included in cluster C3 (DAPC) and q1K3, while q2K4 and q3K4 corresponded to cluster C2 and gene pool q2K3, respectively. The gene pools q2K4 and q3K4 separated genotypes belonging to q3K3, and 89 genotypes were identified as admixed (**Supplementary Table S8**). Genetic relationship analysis supported the clustering with minor differences (**Supplementary Figure S7**). To investigate the genetic differentiation among the four clusters defined by DAPC, pairwise *F*_ST_ values were computed. The analysis showed that genetic differentiation was strong between C3 and C4 versus C1 and C2 (*F*_ST_ > 0.21), high between C1 versus C2 and C3 (*F*_ST_ = 0.14 and 0.13), and moderate between C1 versus C2 (*F*_ST_ = 0.11) and C3 versus C4 (*F*_ST_ = 0.09) (**Figure 6C**).

## 4 Discussion

Nowadays, climate change and its associated conditions have posed significant challenges to olive tree cultivation (Kaniewski et al., 2023). As a result, there is a pressing need for novel approaches to effectively address these challenges and develop adaptation strategies, both in the short and long term, for the effective use of olive genetic resources (D’Agostino et al., 2018). In the present study, a great effort was made to explore the genetic diversity of olive trees germplasm overspread in Jordan, revealing a remarkable richness of genetic diversity, and the potential of this germplasm for various purposes.

Field surveys revealed the extensive distribution of olive trees in a wide range of conditions and habitats. Noteworthy, these olive trees have shown impressive resilience and have thrived in environments ranging from unmanaged farmer’s fields to natural desert habitats. Furthermore, many of these olive trees were in marginal areas, characterized by a broad spectrum of micro-environments and spanning a wide altitude range from 166 to 1220 meters, fluctuating rainfall patterns, and received little or no agricultural interventions (**Supplementary Table S1**). Despite these harsh conditions, these trees have continued to grow and produce high quality yields. The remarkable resilience of the prospected and identified germplasm highlights its value as native genetic material suitable for adaptation to various stresses in harsh environments and future adoption to cope with climate change worse scenarios (Sales et al., 2021). The inclusion and evaluation of such germplasm into *ex situ* germplasm collections and its further use for comparative field trials and in olive breeding programs will potentially expand the narrow genetic base of the current elite olive cultivars in modern olive orchards and improve resilience to climate change (Díez et al., 2015; Arenas-Castro et al., 2020).

In this study, the morphological traits proved to be effective and informative for olive tree discrimination. The morphological characterization of olive trees identified a considerable level of phenotypic variations within the identified genotypes, as shown in the PCA and heatmap analysis (**Figures 2**, **3**; **Supplementary Figure S2**). The analysis allowed the identification of three main groups that formed independently of their geographical origin including the cultivars Kfari Baladi and Kanabisi as well as a diverse set of Jordanian germplasm bank accessions besides Jordanian trees not associated with the Kfari Baladi and Kanabisi cultivars. Similarly, Chalak et al. (2015) identified three main groups of olive trees from Lebanon, which are in general agreement with the results of this study. The PCA analysis of morphological traits indicated that some traits have higher discriminative power between the identified groups. These traits included growth habit, location of fruit color change, fruit and stone diameter, stone termination, and leaf blade curvature. Previous studies confirmed that fruit and stone traits were the most informative traits for olive cultivars identification with a high discriminating power (Trujillo et al., 2014; Titouh et al., 2021; Khadivi et al., 2022). In general agreement with this study, El Bakkali et al. (2019), found that fruit-associated traits were very effective in identifying a total of 251 different morphological profiles in a large olive collection. Stone shape was found to have high discriminating power compared to other traits and can be used to effectively differentiate between major identified groups as previously reported (Veloso et al., 2018; Koubouris et al., 2019). In this study, Kfari Baladi trees had an elliptic stone shape, while Kanabisi had an elongated shape, irrespective to their collection site (**Supplementary Table S2**; **Supplementary Figure S3**). These results agreed with the previous studies, which found that stone traits are less affected by environmental conditions than leaf and fruit traits, due to their high heritability (Terral et al., 2004; Trujillo et al., 2014; D’Agostino et al., 2018). Furthermore, the positive correlation between endocarp and fruit traits are in general agreement with Bazakos et al. (2023). On the other hand, growth habit also proved to be a useful trait for discriminating between olive tree genotypes and particularly between Kfari Baladi and Kanabisi trees, which have an upright growth habit. This may highlight the potential use of Kanabisi trees in intensive farming system due to its upright growth habit as well as its “easy to harvest” features, which make it suitable for mechanized production systems (Tous et al., 2010). Furthermore, Kfari Baladi trees were characterized by medium fruit weight, while Kanabisi and JOGBC accessions had high fruit weight values. In summary, the results of this study highlighted the potential of olive trees in Jordan as a useful genetic resource for key horticultural traits such as fruit weight and growth habit.

Here, informative SNP markers described in previous studies (Biton et al., 2015; Belaj et al., 2018, 2022) were used to develop an effective genotyping assay using the KASP technology (Jatayev et al., 2017). SNPs have proven to be efficient, reliable, and robust molecular markers in the study of the genetic diversity of different plant species, including olive trees (Belaj et al., 2018; D’Agostino et al., 2018; Sion et al., 2021). Recently, 96 EST-SNP markers were used to genotype 1,273 accessions collected from 29 countries in the WOGBC-Cordoba-Spain (Belaj et al., 2022). Despite the high overall genetic diversity, a notable percentage of redundant accessions was observed within and between collected materials, regardless of their countries or regions of origin. In addition, the results of this study highlight that previously tested SNP markers data are reliable and reproducible in discriminating between genotypes, especially when it comes to olives, which have been the subject of numerous studies using various molecular techniques (Marchese et al., 2023; Gómez-Gálvez et al., 2024). Furthermore, this study describes the first use of KASP technology in olive trees, which had proven successful and beneficial in genotyping and discriminating olive accessions. Recently, KASP markers have been used in genetic studies for several fruit trees such as: almond (Goonetilleke et al., 2018), apple (Winfield et al., 2020), and peach (Fleming et al., 2022). The conversion of selected SNPs into KASP markers for olive tree genotyping also proved to be reliable, practical, and cost-effective and their use allowed characterizing the collected olive tree material and discriminating olive accessions. The use of this technology was not only powerful in distinguishing different cultivars, but it also provided a cheaper and automated markers genotyping for used in future olive studies (Winfield et al., 2020).

Using SNP data, the genetic relationship analysis grouped olive trees of Jordan into three main clusters regardless of their geographical origin. This clustering was in general agreement with the results obtained from the analysis of morphological traits. Molecular analysis was able to distinguish between the cultivars Kfari Baladi and Kanabisi and most of the olive trees as well as accessions from the JOGBC. As expected, the JOGBC accessions and most of the sampled trees, with a few regional exceptions shared the same genotype with Kfari Baladi, thus confirming it is a widespread cultivated genotype across the country where it has been named differently. The second cluster included the cultivar Kanabisi and all the trees that displayed the same SNP genotype with this cultivar, indicating that this cultivar is widespread throughout the country and particularly in arid environments of Jordan. The third group of olive trees showed high genetic variation and included accessions from JOGBC and several olive trees from Jordan. The identification of unique genotypes among the prospected trees from regions such as TF, BA, IR, JA and AJ suggests the existence of new and previously uncharacterized genotypes. These findings are in general agreement with previous studies in which it was possible to identify genetically diverse olive trees using molecular markers that were represented only as a small percentage of the dominant olive cultivars (Baldoni et al., 2006; Erre et al., 2010; Gómez-Gálvez et al., 2024). Such autochthonous or native olive tree material represents a genetic reservoir of important alleles, and it is considerably important to increase the percentage of unique genotypes to be collected in hotspot regions (Díez et al., 2011). Furthermore, some of these trees were found to have special agronomic characteristics, especially fruit-related traits, including fruit weight that ranged from medium to very high. These agronomic characteristics are considerably important because they yield larger quantities of olives, which can translate to higher oil production or greater fruit yield for table olives. Furthermore, trees with varying fruit weights may indicate adaptation to different environmental conditions, such as varying levels of rainfall or soil types. Understanding these traits helps in selecting cultivars that are resilient and productive under diverse agronomic settings.

The SNP marker-based cladogram used in this study revealed the presence of redundant genotypes in the collection, that were reported in previous studies (D’Imperio et al., 2011; Mariotti et al., 2020; Belaj et al, 2022). Previous studies using EST-SNP markers identified the largest group of redundant accessions that included the Lebanese cultivar Baladi and 39 identical accessions from six countries including the Sourani cultivar from Syria and several accessions from Jordan (Ketat, Kfari Baladi, Kfari Romi, Arabi Al-Tafilah, and Nabali Baladi). Those results are in total agreement with the molecular data obtained in this study. In the Eastern Mediterranean region, local terminology recognizes four cultivars in traditional olive cultivation: Souri, Nabali Baladi, Nabali Muhassan, and Mallisi, among which Souri is considered the oldest and most predominant variety in the region (Ben-Ari et al., 2014). Genetic diversity of some olive cultivars from the southern Levant using SSR confirmed that the Souri cultivar was highly related to the Romi, Nabali Baladi, Nabali Muhassan, and Mailsi cultivars (Barazani et al., 2023). The second most prevalent cultivar in Jordan, Kanabisi, included trees from desert areas and was identified as synonymous with the Syrian cultivar Safrawi, as along with other cultivars from eight different countries (Belaj et al., 2022). It is interesting to note that Safrawi cultivar alongside Gordal Sevillana cultivar have been considered two main founders of more than 60% of the olive cultivars in the Mediterranean Basin (Mariotti et al., 2023). In another study, the Greek cultivar Throubolia, which is synonymous with the cultivar Safrawi (Belaj et al., 2022) and Kanabisi, showed IBS value of 0.967 with the monumental olive tree Throuba Naxos, estimated to be about 3000 years, linking this cultivar to the early domestication of olive trees in Greece (Bazakos et al., 2023). These results highlight the central role played by Kanabisi in olive domestication in the Mediterranean basin and support the concept of a primary domestication event in the eastern Mediterranean basin followed by a subsequent dispersion towards the West accompanied by secondary domestication events with wild olive populations (Gros-Balthazard et al., 2019; Bazakos et al., 2023).

In this study, the identity of several Jordanian olive germplasms was confirmed by comparing SNP data with WOGBC EST-SNP markers (Belaj et al., 2022). This analysis facilitated the identification and correction of errors within the JGBOC, which is crucial for ensuring the accuracy and reliability of genetic resources for future research. Incorrect labeling or identification of genotypes due to human error can lead to confusion and hinder accurate research and breeding efforts. The implementation of molecular markers for verification can improve record-keeping practices and help in including missing cultivars and wild olive genotypes in gene banks to enhance diversity and prioritize proper conservation methods (Belaj et al., 2022). This will enhance collaboration and information sharing among institutions, thereby contributing to the overall enhancement and sustainability of olive germplasm collections, both within Jordan and globally. The analysis also identified 73 unique and novel genotypes from Jordan that had not been previously reported, highlighting the presence of rich genetic diversity within the country. One of these unique genotypes is represented by as many as six individuals in our collection, suggesting evidence of clonal vegetative propagation, and indicating the cultivated nature of this unknown genotype. The remaining genotypes are considered unique, and this suggests the presence of previously unknown genotypes and potentially novel traits within Jordan’s olive germplasm, underscoring the importance of further exploration and conservation efforts. For instance, a unique olive tree TF-1 produced high fruit weight mean value and flourished in a region where annual rainfall is less than 200 millimetres. In the same region, TF-9 produced very high fruit weight mean value and was closely related to Toffehi, a well-known cultivar with high fruit weight mean value. Besides TF region, high fruit weight has been observed in olive trees from AJ, JA and BA, making them a suitable choice for table olive production. Interestingly, unique olive trees from Borma area (JA) were found in feral status and had small fruit weight mean value when compared with other unique trees from other regions. At the same area, occurrence of wild oleaster is common indicating a hotspot for future collection missions. Furthermore, these unique genotypes exhibited a wide range of phenotypic and genetic variations that can provide a valuable resource for breeding programs aiming to develop olive cultivars adapted to local conditions. It is worth to mention that such unique genotypes could represent previously unrecognized introductions from neighboring regions or as germinating seeds from existing cultivars or even ancient lineages that have persisted in Jordan for millennia (Mousavi et al., 2017). Therefore, future research should focus on understanding the ancestry and genetic relationships of these unique genotypes with genetic material within the Mediterranean basin.

When Jordanian samples were analyzed together with 52 Mediterranean accessions (IOCC-3), they formed distinct clusters separate from Central and Western Mediterranean cultivars, indicating a high degree of genetic diversity unique to Jordan. Similar separation of Eastern Mediterranean cultivars from those in Central and Western regions has been reported previously (El Bakkali et al., 2019; Haddad et al., 2020). In the DAPC analysis, Jordanian olive trees grouped into two clusters distinct from other Mediterranean genotypes. One cluster included 70 trees predominantly from Borma and Maysarah, indicating these locations harbor a hotspot of genetic diversity within Jordan. Furthermore, ancestry-specific allele frequency estimation revealed a high degree of genetic admixture within the Jordanian germplasm. Using a model with four ancestries (K =4), 89 accessions were tagged as admixed, with 60 of these trees originating from Jordan, indicating a complex evolutionary history within the region. Previous studies by Belaj et al. (2010) have observed distinct genetic patterns in both wild olive populations and cultivars in Spain, suggesting regional divergence influenced by local and introduced varieties. This parallels the findings here, suggesting similar dynamics in Jordanian olive populations shaped by historical and modern gene flow. Additionally, Julca et al. (2020) and Zunino et al. (2023) provided lines of evidence of recurrent genetic admixture during domestication, particularly in the Mediterranean Basin. Bazakos et al. (2023) emphasized the prevalence of admixed olive populations in the Western Mediterranean basin and identified distinct genetic clusters within olive populations across the Mediterranean basin, corresponding to different domestication events. These findings are generally consistent with the results of this study, which highlight the complex nature of olive diversity. This diversity is shaped by regional differentiation, domestication processes, and ongoing genetic mixing and evolution, all of which are evident in Jordan, a region considered to be one of the centers of olive domestication. Collectively, these studies highlight the intricate genetic history and emphasize the importance of understanding and conserving olive genetic resources across their native range.

## 5 Conclusions

The study of olive trees in Jordan has revealed their widespread distribution across diverse environments, highlighting their adaptability to different growing conditions. Morphological analysis demonstrated a high degree of diversity in the studied traits among olive tree populations, providing evidence of rich genetic variation within Jordanian olive germplasm. Genetic analysis using informative SNP markers grouped Jordanian olive trees into distinct clades and uncovered potential redundancy among genotypes, particularly within Kfari Baladi and Kanabisi cultivars. Comparative analysis with the Worldwide Olive Germplasm Bank confirmed the reliability of EST-SNP markers and the presence of synonyms, propagation errors and/or mislabeling within the Jordanian olive germplasm collection. These results underscore the critical importance of accurate documentation and in-depth characterization for the effective management and conservation of olive tree genetic resources in germplasm collections as well as *in situ*/on farm. The analysis of population structure resulted into distinct genetic groups, along with the presence of admixed individuals, thus suggesting historical or modern gene flow. Finally, the identification of 73 new olive genotypes in Jordanian environments highlights their potential as genetic resources for future improvement and utilization. These findings remark the urgent need for comprehensive conservation efforts to preserve the rich genetic diversity of Jordanian olive germplasm.

## Data availability statement

The original contributions presented in the study are included in the article/**Supplementary Material**. Further inquiries can be directed to the corresponding author.

## Author contributions

MA-K: Conceptualization, Data curation, Investigation, Methodology, Writing – original draft. FT: Conceptualization, Data curation, Formal analysis, Funding acquisition, Investigation, Methodology, Supervision, Writing – review & editing. ND’A: Data curation, Formal analysis, Methodology, Resources, Software, Writing – review & editing. CM: Data curation, Formal analysis, Methodology, Writing – review & editing. AB: Data curation, Formal analysis, Investigation, Methodology, Validation, Writing – review & editing. SA: Data curation, Methodology, Writing – review & editing. RA: Data curation, Formal analysis, Visualization, Writing – review & editing. SH: Data curation, Methodology, Software, Writing – review & editing. AA-A: Conceptualization, Data curation, Formal analysis, Funding acquisition, Investigation, Methodology, Project administration, Supervision, Writing – original draft.

## Supplementary material

Supplementary Figure 1 Map of Jordan showing the targeted regions for olive trees collection; Capital letters refer to sampling area: **(A)** northern parts (Irbid, Ajloun and Jarash); **(B)** central parts (Balaqa and Gene Bank); **(C)** southern parts (Karak and Tafilah; **(D)** northern parts (Ma’an).

Supplementary Figure 2
**(A)** Biplot showing the distribution of the 24 morphological traits (contrib: the contributions in percentage). **(B)** Biplot showing the distribution of the 24 morphological traits for the 382 olive tress.

Supplementary Figure 3 Morphological characteristics of Baladi and Kanabisi olive trees, fruits, leaves, stones and fruit coloration pattern.

Supplementary Figure 4 Pairwise correlation (Pearson’s coefficients) using 24 phenotypic traits of 382 olive trees identified in this study.

Supplementary Figure 5 Discriminant analysis of principal components (DAPC) of 149 olive accessions. **(A)** The Bayesian information criteria (BIC) supported four distinct genetic groups; **(B)** Variance explained by PCA; barplot of eigenvalues for the discriminant analysis.

Supplementary Figure 6 Plot of ADMIXTURE cross validation error from K=1 through K=15. In red were indicated the two best K (3 and 4), as the value that minimizes the error.

Supplementary Figure 7 Phylogenetic tree constructed using the maximum likelihood method (bootstrapping value of 1000) based on the genotyping data of 45 SNP loci. The analysis included 149 olive trees from JOCC-2, 32 olive trees from JOCC-1 and 34 accessions from JGBOC.
