# Supplementary figures and images for: Evaluation of genetic diversity among olive trees (Olea europaea L.) from Jordan
Source: Front Plant Sci. 2024 Aug 6;15:1437055. doi: 10.3389/fpls.2024.1437055 (PMC11333458; doi:10.3389/fpls.2024.1437055)

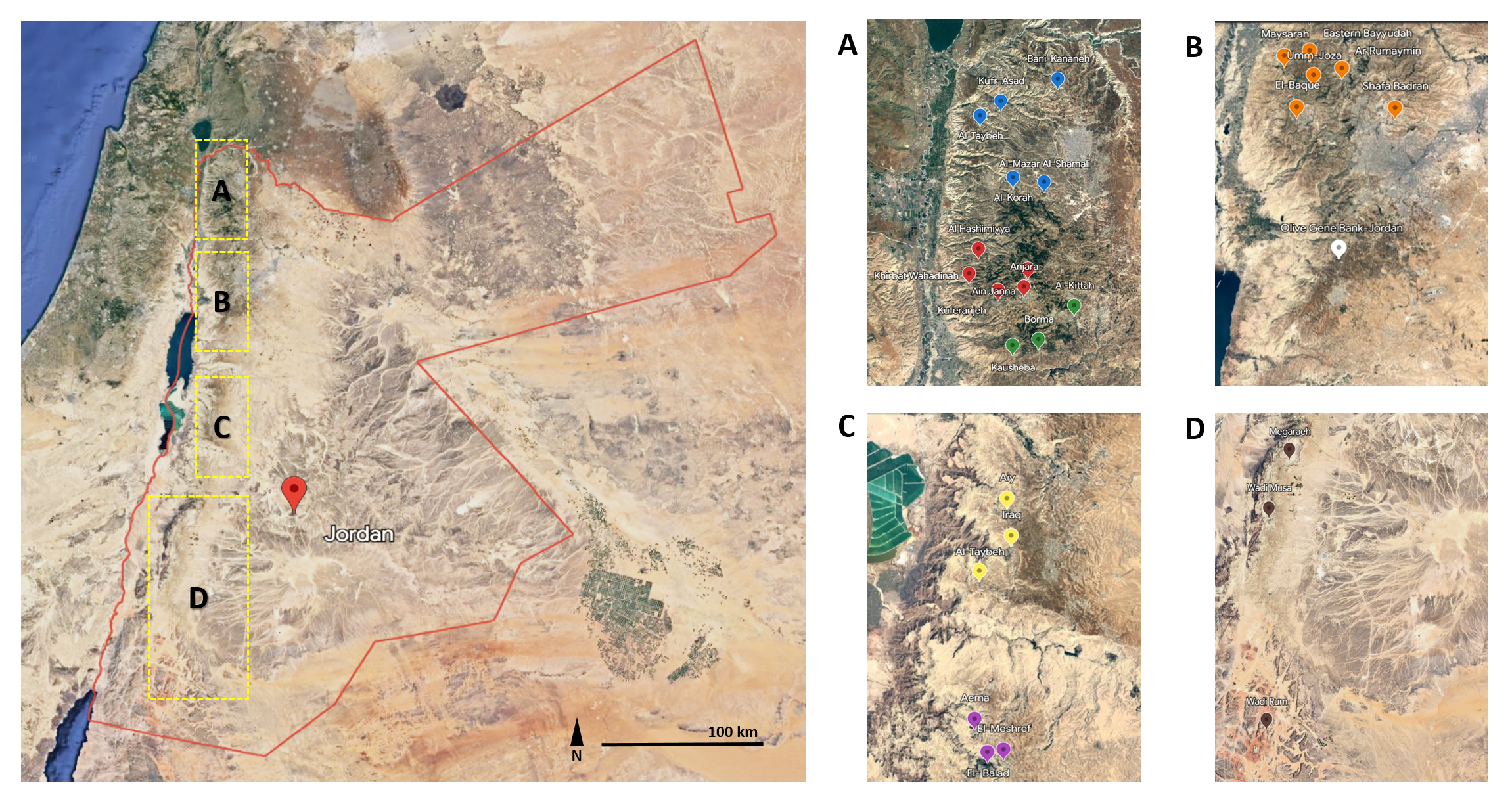

Supplement: Supplementary Figure 1 — Map of Jordan showing the targeted regions for olive trees collection; Capital letters refer to sampling area: (A) northern parts (Irbid, Ajloun and Jarash); (B) central parts (Balaqa and Gene Bank); (C) southern parts (Karak and Tafilah; (D) northern parts (Ma’an). [file Image_1.png]

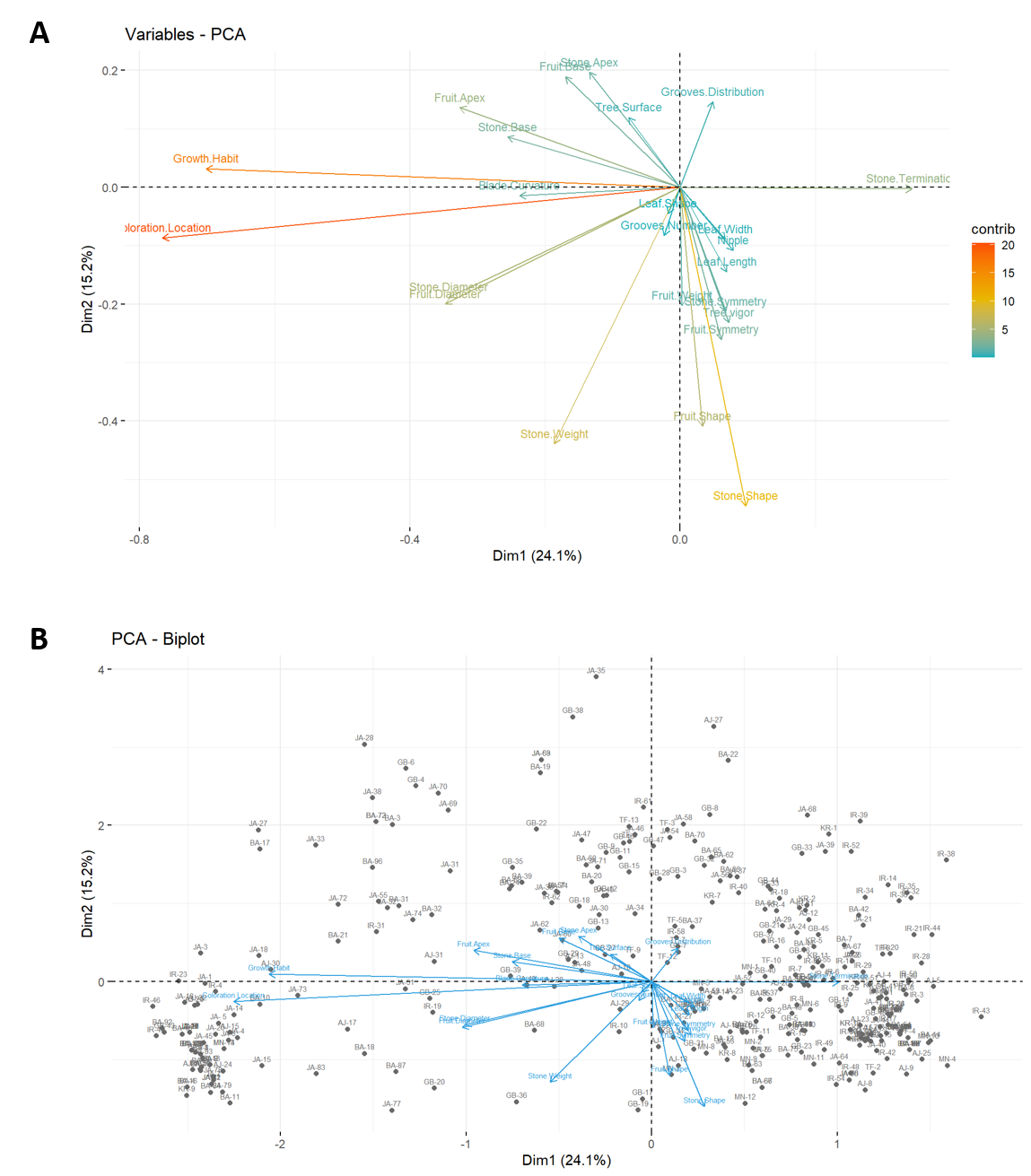

Supplement: Supplementary Figure 2 — (A) Biplot showing the distribution of the 24 morphological traits (contrib: the contributions in percentage). (B) Biplot showing the distribution of the 24 morphological traits for the 382 olive tress. [file Image_2.png]

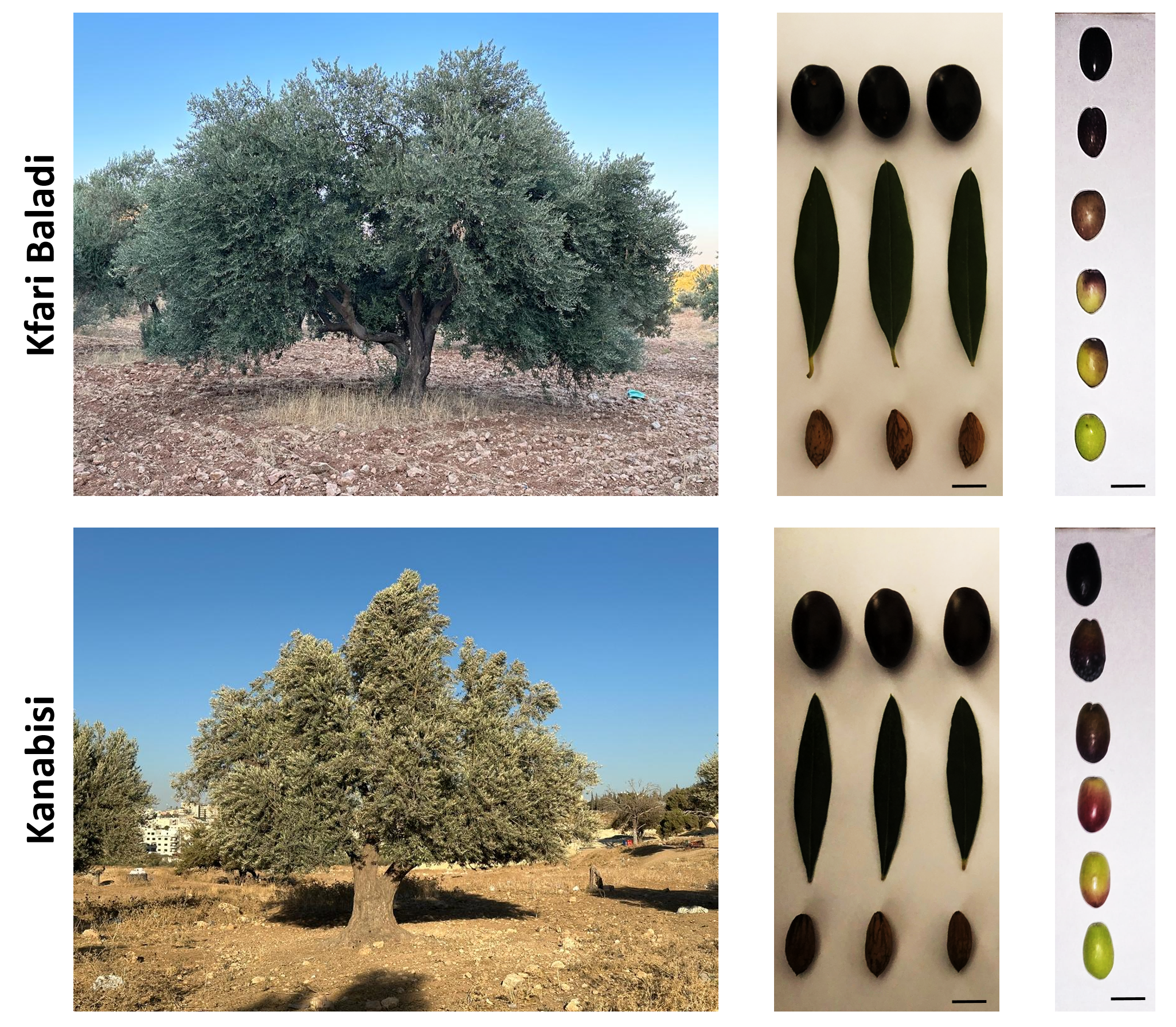

Supplement: Supplementary Figure 3 — Morphological characteristics of Baladi and Kanabisi olive trees, fruits, leaves, stones and fruit coloration pattern. [file Image_3.png]

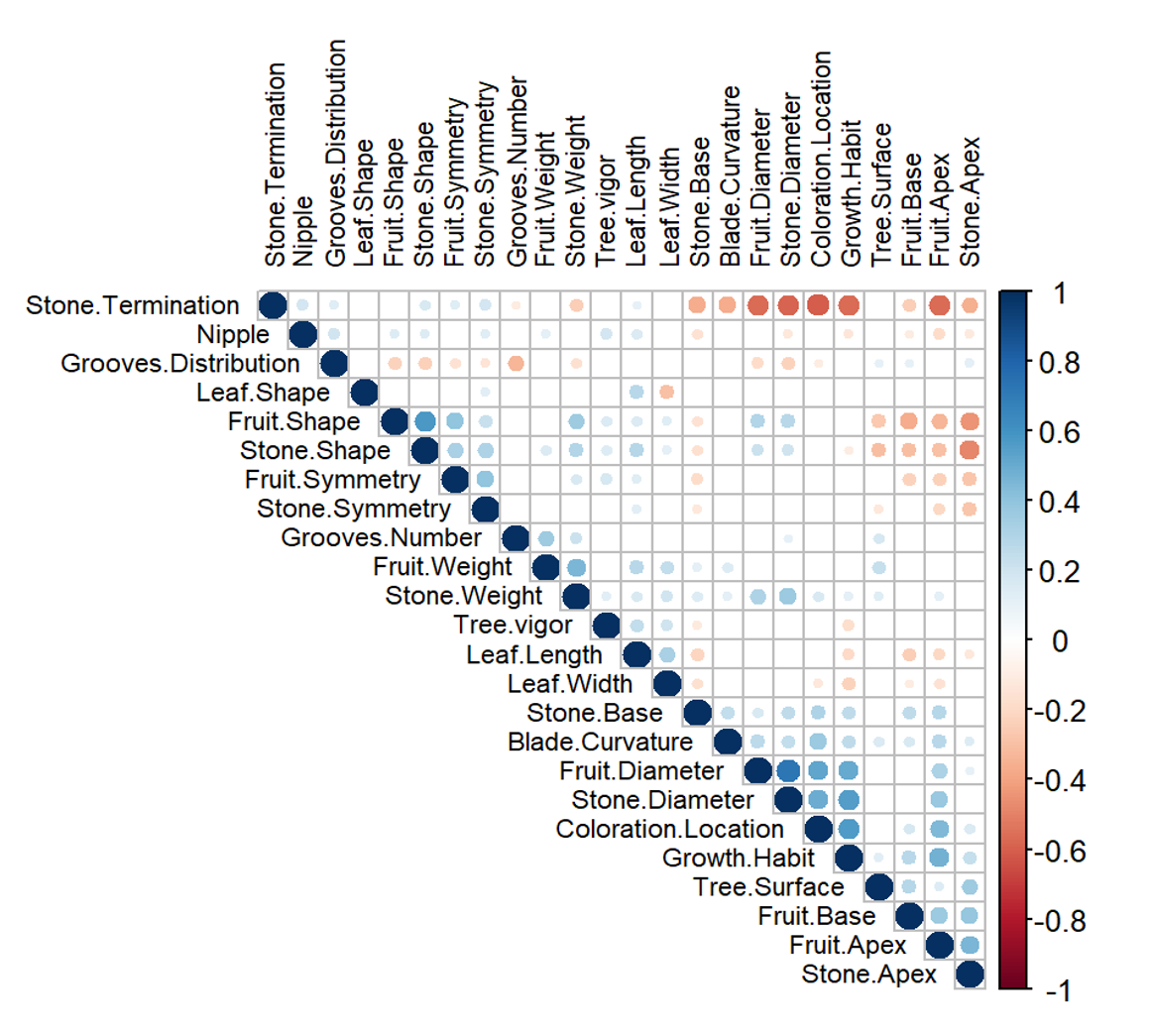

Supplement: Supplementary Figure 4 — Pairwise correlation (Pearson’s coefficients) using 24 phenotypic traits of 382 olive trees identified in this study. [file Image_4.png]

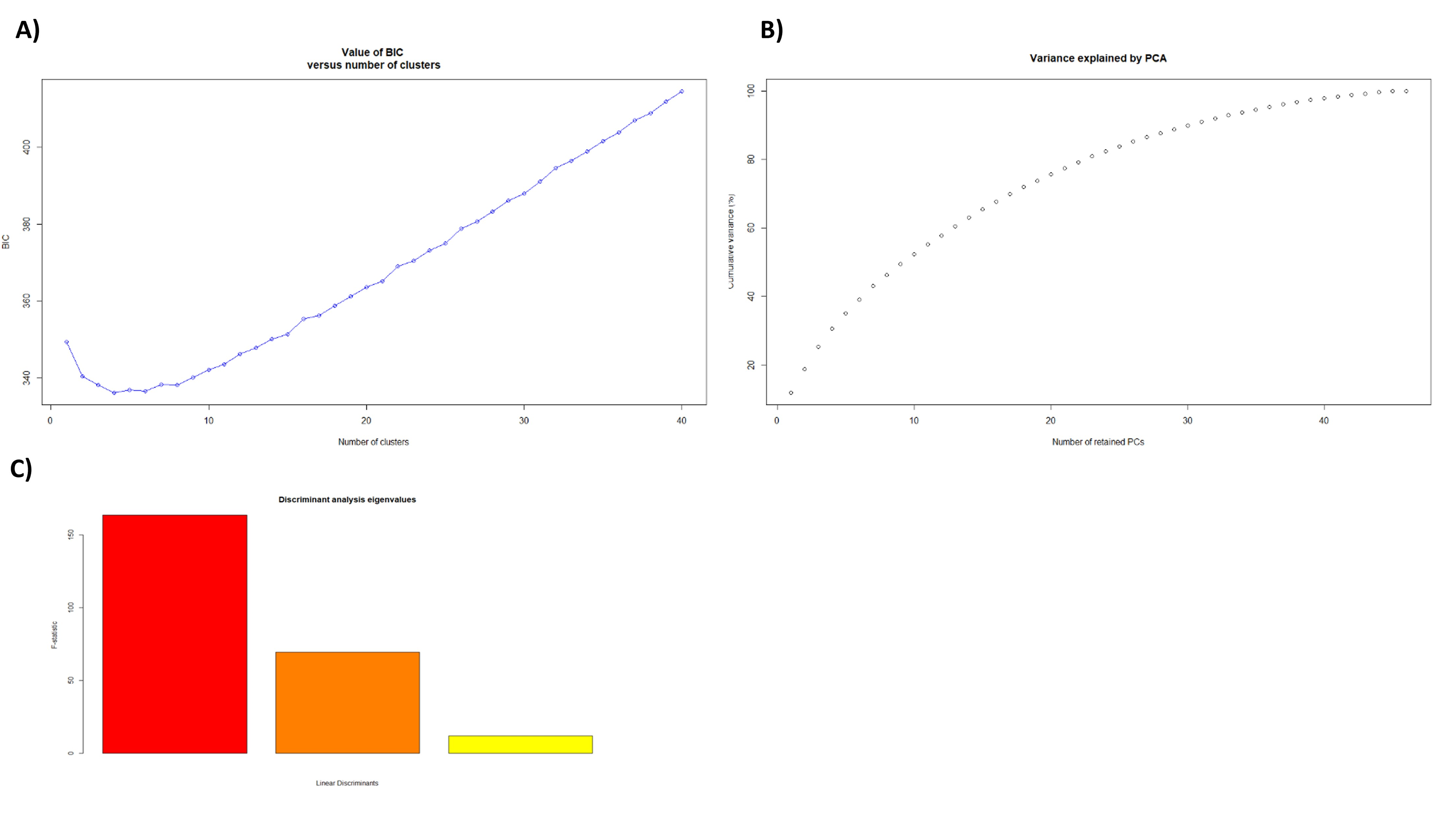

Supplement: Supplementary Figure 5 — Discriminant analysis of principal components (DAPC) of 149 olive accessions. (A) The Bayesian information criteria (BIC) supported four distinct genetic groups; (B) Variance explained by PCA; barplot of eigenvalues for the discriminant analysis. [file Image_5.png]

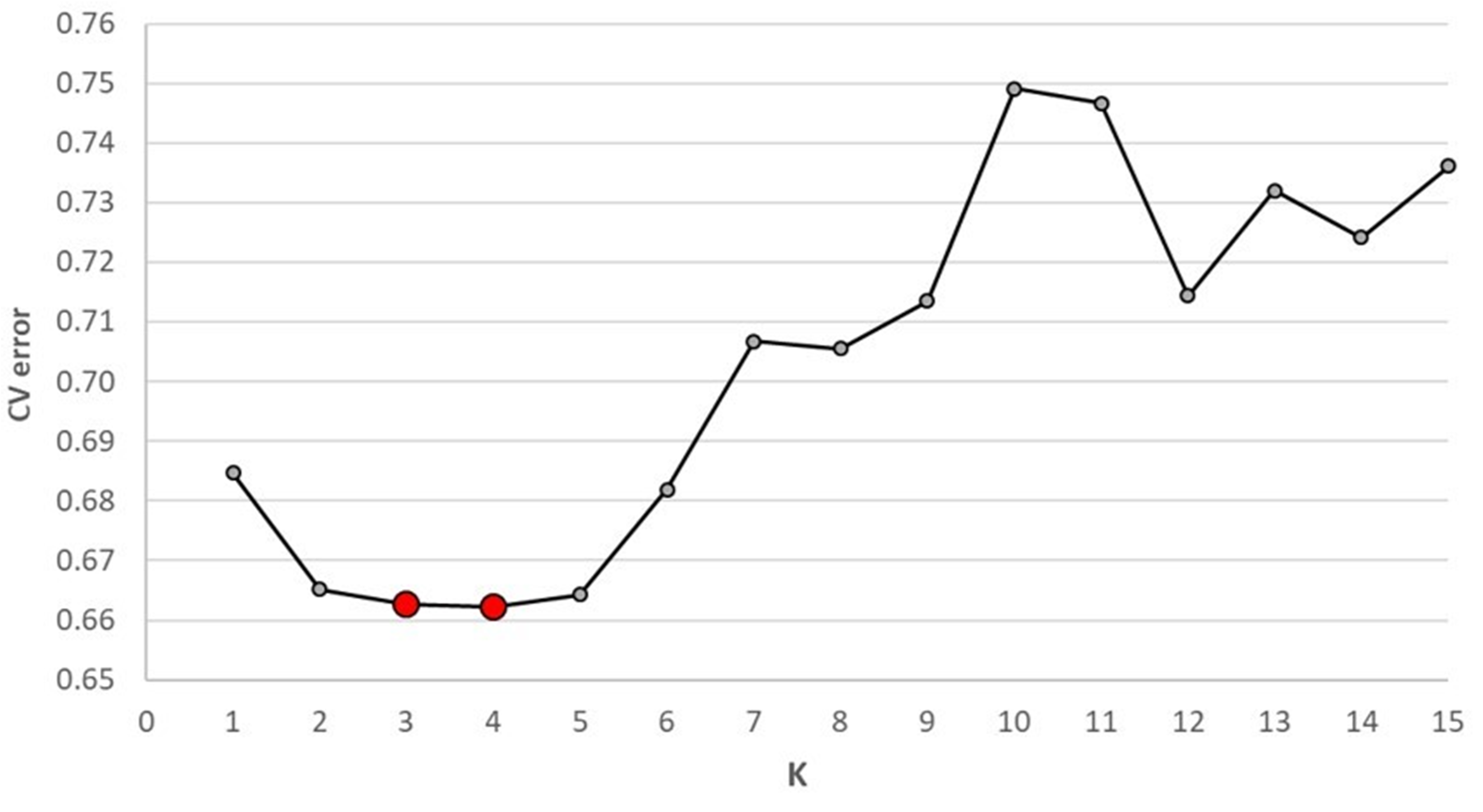

Supplement: Supplementary Figure 6 — Plot of ADMIXTURE cross validation error from K=1 through K=15. In red were indicated the two best K (3 and 4), as the value that minimizes the error. [file Image_6.png]

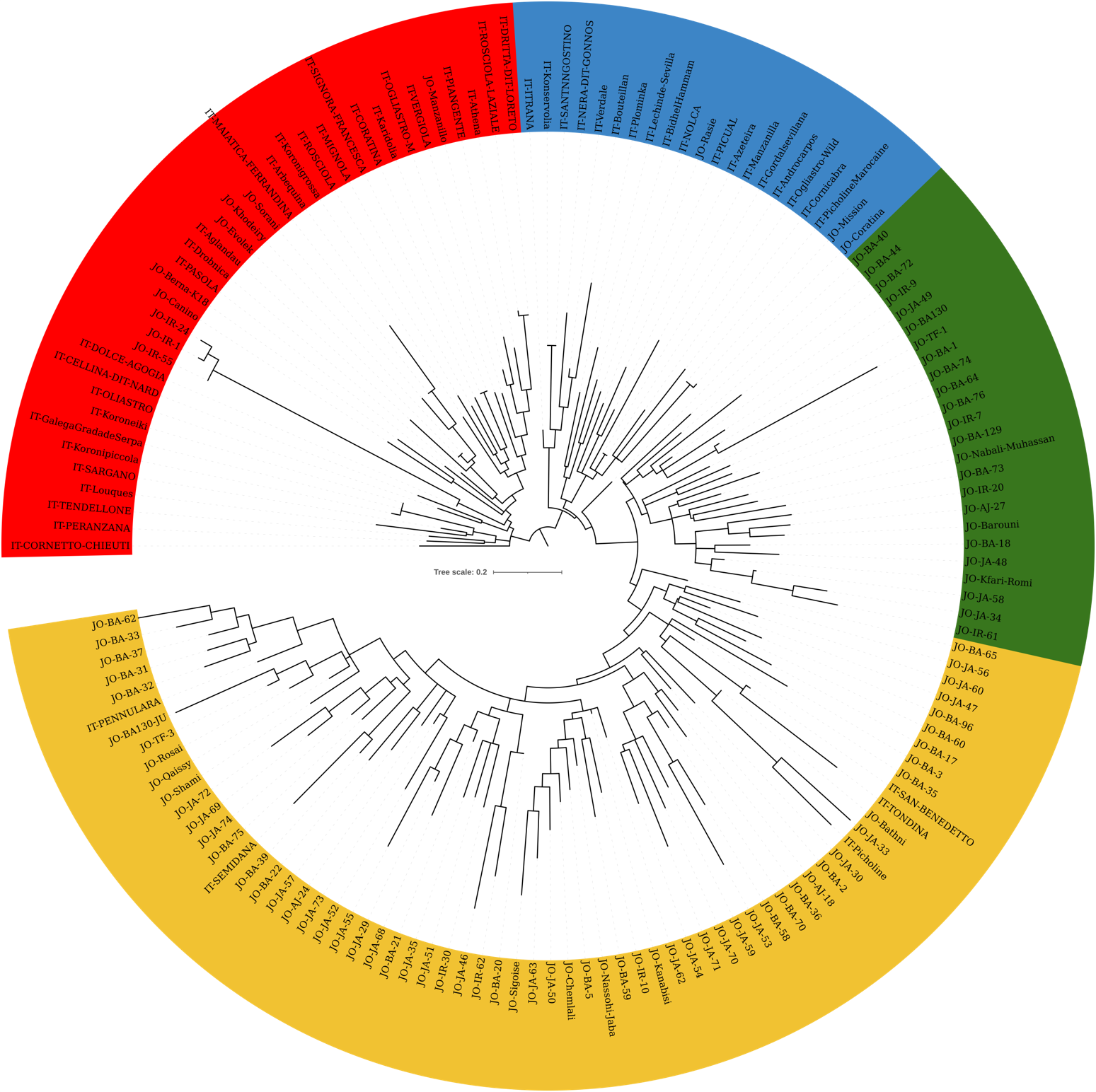

Supplement: Supplementary Figure 7 — Phylogenetic tree constructed using the maximum likelihood method (bootstrapping value of 1000) based on the genotyping data of 45 SNP loci. The analysis included 149 olive trees from JOCC-2, 32 olive trees from JOCC-1 and 34 accessions from JGBOC. [file Image_7.png]
